# Dermatological Neoplastic Diseases Complicating Treatment with Monoclonal Antibodies for Multiple Sclerosis

**DOI:** 10.3390/jcm13175133

**Published:** 2024-08-29

**Authors:** Floriana Bile, Maddalena Sparaco, Eleonora Ruocco, Giuseppina Miele, Elisabetta Maida, Renato Vele, Davide Mele, Simona Bonavita, Luigi Lavorgna

**Affiliations:** 1 Department of Advanced Medical and Surgical Sciences, 2nd Division of Neurology, University of Campania Luigi Vanvitelli, Via Sergio Pansini, 5, 80131 Naples, Italy; floriana.bile@gmail.com (F.B.); maidaelisabetta92@gmail.com (E.M.); renatovele93@gmail.com (R.V.); dott.davidemele@gmail.com (D.M.); 2 2nd Division of Neurology, University Hospital of Campania Luigi Vanvitelli, 80131 Naples, Italy; maddalena.sparaco@policliniconapoli.it (M.S.); giuseppinamiele20@gmail.com (G.M.); 3 Dermatology Unit, Department of Mental and Physical Health and Preventive Medicine, University of Campania “Luigi Vanvitelli”, 80138 Naples, Italy; eleonora.ruocco@unicampania.it; 4 1st Division of Neurology, University Hospital of Campania Luigi Vanvitelli, 80138 Naples, Italy; luigi.lavorgna@policliniconapoli.it

**Keywords:** multiple sclerosis, skin cancer, monoclonal antibodies

## Abstract

**Background:** Over the past 20 years, the treatment scenario of multiple sclerosis (MS) has radically changed, and an ever-increasing number of disease-modifying treatments has emerged. Among high-efficacy treatment agents, monoclonal antibodies (mAbs) have become a mainstay in a MS patient’s treatment due to their targeted mechanism, high efficacy, and favorable risk profile. The latter varies from drug to drug and a skin cancer warning has emerged with sphingosine 1-phosphate receptor inhibitors. Several cases of skin malignancy in people with MS (pwMS) undergoing therapy with mAbs have also been described, but dermatological follow-up is not currently indicated. **Objectives:** The aim of this review is to investigate cases of cutaneous malignancy during mAb therapy and to explore possible pathophysiological mechanisms to evaluate the potential need for regular dermatological follow-ups in pwMS treated with mAbs. **Methods:** A literature search for original articles and reviews in PubMed was conducted with no date restrictions. **Results:** A total of 1019 results were retrieved. Duplicates were removed using Endnote and manually. Only peer-reviewed studies published in English were considered for inclusion. At the end of these screening procedures, 54 studies published between 2001 and 2024 that met the objectives of this review were selected and reported. **Conclusions:** The available data do not show a clear link between monoclonal antibody (mAb) treatment in pwMS and the risk of skin cancer. At present, these treatments remain contraindicated for people with cancer. Dermatological screening is advisable before starting mAb treatment in pwMS, and subsequent follow-ups should be individualized according to each patient’s risk profile.

## 1. Introduction

Multiple sclerosis (MS) is an immune-mediated inflammatory demyelinating and neurodegenerative disease of the central nervous system (CNS) and a leading cause of non-traumatic neurological disability in young adults [1].

In the past 20 years, the treatment scenario of MS has radically changed, and an ever-increasing number of disease-modifying treatments (DMTs) has emerged, enabling the effective reduction of disease activity, disability accrual, and accumulation of irreversible damage by interfering with a variety of immunological mechanisms [2].

DMTs can be classified into drugs of low- or moderate-efficacy agents (interferons, glatiramer acetate, teriflunomide, dimethyl fumarate) and high-efficacy treatment agents (HETA) that comprise sphingosine 1-phosphate (S1P) modulators, cladribine, and monoclonal antibodies (mAbs) [3]. Treatment allocation is driven by an individualized evaluation of the risk–benefit profile, according to disease activity, disease phenotype, and the patient’s needs. mAbs have become a mainstay in an MS patient’s treatment due to their targeted mechanism, high efficacy, and favorable risk profile [2].

mAbs can be classified according to their molecular structure: the first generation of therapeutic mAbs was developed from non-human species such as mice (fully murine). To reduce the potential immunogenicity of murine mAbs, chimeric mouse–human, humanized mAbs, and then fully human mAbs were implemented [4].

The first mAb introduced for MS treatment was Natalizumab (NTZ), an antibody against the α4 subunit of human integrins, which inhibits the transmigration of lymphocytes across the blood–brain barrier. In contrast, Alemtuzumab (ALZ) and the class of anti-CD20 mAbs (such as ocrelizumab and ofatumumab) act as depletors of a specific class of blood cells [2].

The safety profile of HETA varies from drug to drug. In particular, a warning for cutaneous cancers emerged with Fingolimod, a S1P receptor inhibitor that sequesters lymphocytes in lymph nodes and secondary lymphoid tissues. In detail, although the most common cutaneous malignancies in people with MS (pwMS) treated with Fingolimod are basal-cell carcinoma and Bowen disease [5], cases of melanoma have also been reported [6]. These data highlighted the importance of dermatological screening and monitoring in pwMS treated with Fingolimod. Several cases of skin malignancy have also been described in pwMS undergoing therapy with mAbs, but dermatological follow-up is not currently indicated. The aim of this literature review is to investigate cases of cutaneous malignancies during therapy with mAbs and to explore possible pathophysiological mechanisms to potentially establish the need for regular dermatological follow-ups in pwMS treated with mAbs.

## 2. Materials and Methods

The literature search was conducted using PubMed, Scopus, and Google Scholar as electronic databases. Studies were identified using a combination of the following medical subject headings (Mesh) terms: “Multiple Sclerosis”; “Melanoma”; “Basal-cell carcinoma”; “Squamous cell carcinoma”; “Skin cancer”; “Skin neoplasm”; “Skin tumor”; “Cancer risk”; “Skin malignancy”; “HETA”; “DMT”; “Highly effective DMT”; “Safety”; “monoclonal antibodies”; “Natalizumab”; “Ocrelizumab”; “Ofatumumab”; and/or “Alemtuzumab”. No date restrictions were used. The reference list was crafted by assessing its pertinence to the themes addressed in this review. The following inclusion criteria were applied: (1) papers focusing on the safety data associated with the use of mAbs for the treatment of MS with reference to dermatological malignant complications; and (2) papers exploring possible links between the pharmacodynamic properties of mAbs and the underlying pathophysiology of tumor development and/or progression. The following exclusion criteria were applied: (1) articles focusing on DMTs other than mAbs; (2) articles not dealing with the dermatological neoplastic complication of HETAs used in MS; (3) non-neoplastic dermatological findings and benign neoplastic lesions associated with mAb treatment; and (4) books and letters to editors.

## 3. Results

A total of 1019 results were retrieved. Duplicates were removed using Endnote (online version) and manually. All articles that did not match the title profile were discarded. Only peer-reviewed studies published in English were considered for inclusion. At the end of these screening procedures, 54 studies published between 2001 and 2024 that met the objectives of this review were selected and reported. Specifically, only articles that addressed safety issues related to mAbs used in the treatment of MS, with a focus on dermatological neoplastic complications, were included.

### 3.1. Natalizumab (NTZ)

NTZ is a humanized mAb (IgG4k) that targets the α4 subunit of human integrins (α4/β1 integrin-CD49d/CD29-very late antigen-4 VLA-4), adhesion molecules that are expressed at high levels on the membrane of all leukocytes except neutrophils [7]. NTZ blocks α4β1 integrin interaction with VCAM-1 (or CD106), a vascular cell adhesion molecule, and inhibits leukocyte transmigration across the blood–brain barrier, thereby reducing inflammation in the CNS. NTZ may also exert its anti-inflammatory effects by blocking leukocytes binding to other endothelial components, such as fibronectin and osteopontin, which modulate the survival, priming, and activation of white blood cells that have gained access to the CNS parenchyma [8]. In addition, by binding to the α4/β 7 integrin, NTZ prevents its interaction with the endothelial cell adhesion molecule receptor (MadCAM-1). Therefore, in pwMS, anti-VLA-4 treatment reduces the lymphocyte count in cerebrospinal fluid and the relapse rate [9].

From data sheets, the most common adverse events associated with anti-VLA-4 treatment include headache (32%), nasopharyngitis (27%), fatigue (23%), urinary tract infection (16%), nausea (15%), arthralgia (14%), and dizziness (11%) [10]. Among the most serious side effects, progressive multifocal leukoencephalopathy (PML), an opportunistic infection caused by the JC virus, is a less common but frightening complication that can be fatal or severely disabling [11]. NTZ efficacy and safety in patients with relapsing–remitting (RR) MS has been evaluated in a two-year phase 3 clinical trial, the “Natalizumab Safety and Efficacy in Relapsing Remitting Multiple Sclerosis” (AFFIRM) study [12]. In the AFFIRM trial, five cases of cancer were reported in a group of pwMS treated with NTZ (three cases of breast cancer, one case of stage 0 cervical cancer, and one case of newly diagnosed metastatic melanoma) [12].

The long-term safety and efficacy of NTZ in RRMS patients has been evaluated in the Tysabri Observational Program (TOP), an open-label, multinational, prospective, observational study, including data from July 2007 to November 2017 [13]. Notably, the rates of development of malignancies remained very low: 66 out of 6148 enrolled pwMS (1.1%), with 39 different types of malignancy. The most common malignancy was breast cancer (in 19 pwMS). The dermatological malignancies detected in the study were melanoma in situ (two cases), choroidal melanoma (one case), basal cell carcinoma (one case), and lentigo maligna (one case).

The Southern Network on Adverse Reactions (SONAR), a National Cancer Institute-funded pharmacovigilance program, found an association between NTZ treatment and the risk of melanoma [14]. Data sources included adverse events reported to the Food and Drug Administration (FDA) and peer-reviewed publications. In particular, the FDA Adverse Event Reporting System (FAERS) described 137 reports of NTZ-associated melanoma. The median age at diagnosis of melanoma was 45 years. A total of 16% developed from pre-existing nevi. In 34% of pwMS, melanoma was diagnosed within 2 years of starting NTZ.

Analyzing data from the FAERS, EudraVigilance (European Medicines Agency), and the Northwestern Medicine Enterprise Data Warehouse (NMEDW), the Research on Adverse Drug Events And Reports (RADAR) program confirmed the SONAR findings [15].

Conversely, Castela et al. showed no increased rate of clinical and dermoscopic changes in 248 pigmented lesions in 44 pwMS treated with NTZ during a prospective follow-up of 14 months [16]. Pharaon et al. confirmed this finding in 74 pwMS with 775 melanocytic skin lesions monitored for more than 4 years. No melanoma was diagnosed. In addition, in vitro analysis of moles removed during the follow-up of NTZ-treated MS patients showed a reduced expression of secreted acidic cysteine-rich protein (SPARC) and β3 integrin on melanocytic cells, proteins that are normally able to promote melanoma invasiveness. Furthermore, a combined in vitro approach was performed to analyze the effects of NTZ on cultured melanoma cells, which demonstrated anti-invasive and anti-migratory properties of the drug in a dose-dependent manner [17].

Alping et al. evaluated the risk of cancer in 6136 pwMS treated with rituximab, NTZ, and Fingolimod, compared with 37,801 subjects from the non-MS general population. Of 1670 pwMS treated with NTZ, only two developed melanoma and no case of other skin cancer was reported [18].

The “Tysabri global observational program in safety” (TYGRIS) study evaluated the risk of cancer during 5 years of follow-ups in 6434 enrolled pwMS who received at least one dose of NTZ [19]. Basal cell carcinoma was reported in 10 pwMS and melanoma in 13, excluding one case of metastatic melanoma without an identified primary site on the skin. Melanoma in situ was present in five of these cases. Overall, when these results were compared with the Surveillance, Epidemiology and End Results (SEER) (for the U.S. general population) and with GLOBOCAN (for the European general population) rates, there was no evidence of an increased risk of melanoma in pwMS treated with NTZ.

### 3.2. Anti CD20 mAbs

The class of anti-CD20 monoclonal antibodies that are routinely used in the treatment of MS includes ocrelizumab (OCR) and ofatumumab (OFA).

OCR is a humanized, intravenously administered mAb that eliminates B cells through antibody-dependent cellular cytotoxicity, complement-dependent cytotoxicity, phagocytosis, and antibody-dependent cellular apoptosis, sparing B cell reconstitution capacity, pre-existing humoral immunity, and innate immunity [20].

Infusion reactions (such as itching, rashes, and difficulty breathing) and infections are the most commonly reported side effects of OCR. According to European public assessment reports (EPAR), OCR may increase the risk of malignancies (e.g., breast cancer) and its use is therefore contraindicated in patients with known malignancies in the European Union [21].

The efficacy and safety of OCR in pwMS have been evaluated in three pivotal phase 3 trials called OPERA I and OPERA II (for pwRRMS) and ORATORIO (for pw primary-progressive (PP) MS) [22,23].

More specifically, OPERA-I and OPERA-II were phase 3, multi-center, randomized, double-blind trials that investigated the efficacy and safety of ocrelizumab compared with subcutaneous interferon beta-1a in pwRRMS. The groups of patients treated with OCR consisted of 410 patients with MS in the OPERA I study and 417 patients with MS in the OPERA II study. Among the adverse reactions reported during the 96-week study period, four malignant tumors were reported in the two OCR groups and only one was a malignant melanoma. Five more malignancies were reported during the open-label extension phase, two of which were basal cell skin tumors and one malignant melanoma [22]. The ORATORIO study, a phase 3, randomized, double blind, placebo-controlled trial, investigated the efficacy and safety of OCR in pwPPMS. Of the adverse reactions reported in the 486 patients enrolled in the OCR arm, 11 were neoplasms, including three basal cell carcinomas. Two additional cases of neoplasia were reported during the open-label extension phase: one case of basal cell carcinoma and one case of squamous cell carcinoma [23].

CASTING is a phase 3, open-label, single-arm study designed to evaluate the efficacy of OCR in pwRRMS with a previous suboptimal response to one or two DMTs. In terms of safety outcomes, only one case of basal cell carcinoma was reported among the 680 pwMS patients treated with OCR [24].

Nevertheless, safety analyses based on integrated clinical and laboratory data from all patients who received OCR in 11 clinical trials, including the controlled treatment and open-label extension (OLE) periods of the phase 2 and 3 trials, plus the phase 3b trials (VELOCE, CHORDS, CASTING, OBOE, ENSEMBLE, CONSONANCE, and LIBERTO), suggest long-term tolerability and no increased risk of malignancy or female breast cancer compared with matched reference MS and general populations or a time-dependent exposure effect [25]. Specifically, the standardized rate of non-melanoma skin cancer (0.20 per 100 patient years [PY] of exposure [95% CI 0.11–0.38]) remained constant and in line with epidemiological benchmarks (0.19 per 100 PY [95% CI 0.15–0.24]).

Smoot and colleagues conducted a prospective cohort study of 355 MS patients treated with OCR from the Providence Ocrelizumab Registry. Of these patients, 32 (9%) had a history of cancer, and eight patients were subsequently diagnosed with cancer after starting OCR: four with basal cell carcinoma, two with breast cancer, one with recurrent thyroid cancer, and one with melanoma [26].

These results align with observations from other anti-CD20 treatments, such as rituximab, which do not show a propensity for primary malignancies [18]. Ongoing post-approval safety studies (CONFIDENCE, MANUSCRIPT, and VERISMO) involving approximately 9000 pwMS newly treated with OCR aim to provide insights into long-term safety, particularly assessing the potential risk of malignancies [25,27,28]. Weber and colleagues presented data from the two-year interim analysis of the CONFIDENCE study, which included 1702 patients with RRMS and 398 patients with PPMS, that have been treated with ≥1 dose of OCR. Ten malignancies were reported: seven occurred in patients with RMS: two cases of malignant melanoma, one case each of basal cell carcinoma, bronchial carcinoma, and thyroid cancer. Three patients with PMS had neoplasms, all cutaneous: a malignant melanoma, a squamous cell carcinoma of the skin, and a basal cell carcinoma [29].

Consistent with the previous literature, an international retrospective pharmacovigilance disproportionality analysis performed on the WHO pharmacovigilance database, VigiBase^®^, from 1 January 2000 to 1 September 2019, found no evidence of an association between OCR and a risk of skin cancer [30,31,32].

OFA is a subcutaneous administered, fully human, anti-C20 mAb approved for RRMS. OFA binds a small exposed extracellular loop epitope of the CD20 receptor, distinct from that recognized by rituximab and OCR, and depletes B cells via complement-dependent cytotoxicity and, to a lesser extent, antibody-dependent cell-mediated cytotoxicity. For this reason, OFA has a higher affinity for B cells and higher potency compared to the aforementioned anti-CD20 mAbs and is able to achieve almost complete B cell depletion at a lower concentration [33]. OFA has been tested in adult RRMS patients in the phase 3 ASCLEPIOS I and II trials and has shown a favorable risk–benefit profile compared to teriflunomide in the broad RRMS population. In these phase 3 trials, five neoplasms (0.5%) occurred in the OFA group, three of which were cutaneous (two basal cell carcinomas and one melanoma) and four (0.4%) in the teriflunomide group (two cases of basal cell carcinoma and one case each of cervical carcinoma and fibrosarcoma) [34]. Preliminary results from the ongoing extension study (ALITHIOS) indicate that RRMS patients undergoing long-term treatment with OFA for up to 4 years show good tolerability, with no new safety concerns detected. The occurrence of malignancies was minimal, as indicated by the overall safety analysis set, with an incidence of 0.86% and an estimated annualized incidence rate (EAIR) of 0.33 (95% CI: 0.20–0.53), similar to that observed during the core period of the ASCLEPIOS I/II OFA arm (incidence of 0.53% and an EAIR of 0.32 (95% CI: 0.13–0.77)) [35].

### 3.3. Alemtuzumab (ALZ)

ALZ is a humanized IgG mAb that binds the CD52 antigen, a cell surface glycoprotein, expressed by T and B lymphocytes, monocytes, macrophages, eosinophils, natural killer cells, thymocytes, and the male reproductive system. ALZ causes cell lysis and the depletion of T and B lymphocytes via complement fixation, antibody-dependent cell-mediated cytotoxicity, and the induction of apoptosis [36].

ALZ has its most pronounced impact on cell immunity, leading to profound and extended lymphopenia, primarily affecting CD4+ lymphocytes, although the deficiency is never entirely exclusive. Indeed, ALZ influences B lymphocytes, resulting in a reduction in the count of circulating antibodies [37].

A specific pattern of lymphocyte repopulation and a shift in cytokine expression toward a less inflammatory profile follows cell depletion, leading to a durable efficacy; B cell and monocyte counts typically normalize within three months. In contrast, T cell counts are suppressed for a longer period, with an average recovery time of 30 months for CD8+ and 60 months for CD4+ to return to pre-treatment levels. ALZ is used in the treatment of B cell malignancies, as induction therapy for solid organ transplantation, in advanced cases of mycosis fungoides, and in various hematological disorders. In the European Union, ALZ is authorized for use in adults diagnosed with active RRMS, as defined by clinical or imaging features [37].

The efficacy and safety of ALZ in pwRRMS have been evaluated in three pivotal studies: Phase II (CAMMS223) and Phase III “Comparison of Alemtuzumab and Rebif Efficacy in Multiple Sclerosis 1 and 2” (CARE-MS and CARE MS-II) [38,39,40]. In phase 3 clinical trials, ALZ and high-dose s.c. interferon-beta-1a were compared in treatment-naïve RRMS patients (CARE-MS I) and in RRMS patients with breakthrough disease who were receiving platform therapies (CARE-MS II). The most common adverse events observed in clinical trials and post-marketing experience included infusion-associated reactions (IARs), such as pyrexia, chills, rash, urticaria, and dyspnea, increased risk of infections and autoimmune disorders such as thyroid disorders, autoimmune thrombocytopenia or other cytopenias (such as neutropenia or pancytopenia), and autoimmune nephropathies. Preliminary data in pwMS do not suggest an association between ALZ and an increased incidence of any cancer, including skin cancer. Overall, in all clinical trials, 29 out of 1486 patients treated with ALZ developed malignancies, including six basal cell carcinomas and four melanomas. In summary, neoplasms were not statistically more frequent with ALZ than with the comparator interferon-beta-1a [38,39,40]. These findings were confirmed by a registry-based cohort study of the entire Danish MS population receiving ALZ between 2009 and 2019, with a total of 209 enrolled patients [41].

A case of malignant transformation of a melanocytic naevus following treatment with ALZ was reported by Pace et al. in 2008 [42]. The patient was a 34-year-old woman with aggressive relapsing MS, previously treated with beta-interferon (stopped two years earlier), who received 120 mg of ALZ divided over five consecutive days, with no immediate side effects apart from a transient cutaneous rash. Six months after treatment, she was diagnosed with a superficial spreading melanoma in the vertical growth phase, arising from a long-standing melanocytic naevus on her back. The lesion had a mild lymphocytic infiltrate. Two other cases of melanoma have been reported in a retrospective study of Austrian patients (*n* = 100) treated with ALZ for B cell chronic lymphocytic leukemia [43].

Registration extension studies and pharmacovigilance analyses seem to refute the association between ALZ and an increased risk of skin cancer.

Results from an extension study (NCT00930553) in patients who received ALZ during the core CARE-MS showed a total of six malignancies over 5 years. Two malignancies occurred in the core study, while four malignancies were reported in years 3–5. Of these, only one malignancy affected the skin, specifically keratoacanthoma [44].

The CAMMS03409 and TOPAZ extension studies assessed the efficacy and safety of ALZ over 12 years of follow-ups; in these extension studies, out of a total of 60 patients, only two developed malignancies: all were melanomas and occurred during the 10th year of follow-ups. A patient with a family history of melanoma developed two grade 4 malignant melanomas, which the study investigator deemed possibly related to the study drug. The second patient was diagnosed with grade 2 melanoma in situ on the abdomen, a condition that the investigator considered unrelated to the study drug and was resolved by surgical excision [45,46].

An observational, cross-sectional, pharmacovigilance cohort study examined individual case safety reports from the WHO database (VigiBase^®^) and found no association between ALZ and cancer reporting [32].

## 4. Discussion

This review summarizes the cases of skin malignancy during therapy with mAbs, both during pivotal trials, observational studies, and pharmacovigilance open data (Table 1).

### 4.1. Natalizumab (NTZ)

Among the mAbs used in MS, the largest available data concern skin malignancies reported in treatment with NTZ. In the European Union, NTZ is indicated for adults with highly active RRMS [10]. NTZ targets the α4 subunit of the VLA-4, in particular, it blocks α4β1 integrin interaction with VCAM-1 [7]. VLA-4 is an integrin expressed at high levels on the membrane of all leukocytes, except neutrophils, that plays an important role in melanocyte cell homeostasis. Integrins constitute a large family of heterodimeric transmembrane glycoproteins responsible for mediating cell–cell and cell–environment interactions. Integrins consist of two subunits: the α-subunit, ranging in size from 120 to 170 kDa, and the β-subunit, measuring 90–100 kDa. In humans, there are 18 α-subunits and eight β-subunits that can combine to form 24 different integrins, each characterized by unique binding properties, tissue distribution, and biological functions. Notably, integrin β1 is the most abundantly expressed integrin subunit. It forms heterodimers with at least 12 α-subunits, giving rise to 12 different isoforms [47].

A member of this family is the very late activation antigen-4 (VLA-4, α4β1), which is expressed under physiological conditions in various leukocyte subtypes. It is also identified on melanoma, osteosarcoma, and rhabdomyosarcoma cells [48].

Several studies suggest that VLA-4 expression on melanoma cells may promote metastatic spread of the tumor. VLA-4 can interact with its ligand, vascular cell adhesion molecule-1 (VCAM-1), which is expressed by activated endothelium. This interaction mediates adhesion and facilitates the subsequent transmigration of tumor cells. In contrast to benign melanocytic lesions, malignant melanomas exhibit an elevated expression of VLA-4, enabling tumor cell migration through the vascular system into any tissue in which endothelial VCAM-1 is expressed. Therefore, the increased expression of VLA-4 on melanoma is correlated with an unfavorable clinical outcome and, if so, inhibition of α4β1 integrin could prevent the spread of metastases [48].

Moreover, there is some evidence that the α4β1 integrin is involved in the lymph node dissemination of melanoma cells [47]. Specifically, α4β1 expressed on tumor cells mediates their binding to lymphatic endothelial cells (LECs) via VCAM-1. In addition, the lymphangiogenic growth factor VEGF-C, secreted by tumor cells and transported in the extracellular matrix, appears to promote the expression and activation of α4β1 integrin on LECs, while the inhibition of α4β1 in LECs appears to significantly prevent lymphangiogenesis at the tumor periphery and the formation of lymph node metastases [47]. Many studies have pointed out that tumor lymphangiogenesis plays a role in promoting lymphatic metastases, providing a direct conduit for tumor cells to escape to nearby draining lymph nodes. In addition, the overexpression of integrin α4β1 on primary melanoma cells appears to be associated with increased bone metastasis, probably through the interaction with VCAM-1, which is constitutively expressed on bone marrow stromal cells [49].

While several studies suggest that VLA-4 expression may promote melanoma metastasis, Qian et al. showed that the overexpression of the cell surface integrin α4β1 prevented the invasion of highly metastatic melanoma cells by facilitating homotypic intercellular adhesion, without having a significant effect on tumorigenicity and tumor cell growth [50]. Therefore, there is a concern that an antibody such as NTZ that binds to α4 may influence melanoma cell replication, invasion, and migration at the cellular level. Recent evidence suggests that the effect of NTZ may vary depending on the drug dose and the specific melanoma cell line [51]. In particular, studying in vitro three different human melanoma cell lines (LCP-Mel, GR-Mel, and WM115) derived from primary tumors, Carbone et al. found that, regarding GR-Mel and LCP-Mel, cell migration significantly augmented after treatment with NTZ. On the other hand, the WM115 cell line had a lower migration rate upon treatment with NTZ [50]. Furthermore, NTZ could affect melanoma development and progression through different mechanisms; one of these may be interference with innate immunity.

Several studies have demonstrated the significant role of natural killer cells (NK) in the defense against melanoma [52]. NK cells, as effector lymphocytes in the innate immune system, play a crucial role in controlling diverse tumors and microbial infections through a dual mechanism. This involves both cytotoxic functions and the production of cytokines. The activation of NK cells is regulated by an elaborate receptor system that includes several cell surface-activating and inhibitory receptors. This system enables NK cells to differentiate between normal cells expressing MHC class I and diseased cells lacking MHC class I molecules on the surface, a phenomenon observed in neoplastic cells, including melanomas. VLA-4 is expressed on NKs and is involved in their migration across endothelial membranes. The observation that the blockade of VLA-4 reduces NK cytotoxicity and modulates crosstalk with melanoma cells suggests that VLA-4 plays a role not only in NK cell adhesion and migration across the endothelial barrier, but also as an activating signal in the key functions of these cells. Moreover, studies have shown that prolonged in vitro treatment of human NK cells expressing integrin α4β1 with inhibitors such as NTZ led to a decrease in NK cell degranulation as well as reduced NK cell migration toward melanoma cells. Finally, α4β1 integrin expression was reduced by NTZ treatment both in vitro and in pwMS, and decreased with the duration of NTZ therapy, suggesting that this drug may alter NK-mediated immune surveillance against melanoma with a protumorigenic outcome [52].

Kimura et al. have shown that NTZ, as an α4 antagonist, can act on different types of T lymphocytes expressing this antigen (TH1, TH17, and T-reg), but appears to exert its effect primarily on T-reg lymphocytes, reducing their circulating levels and disrupting the balance between inflammatory and regulatory T cells in the CD49d+ population [53].

T-regs play a crucial role in maintaining immune balance and preventing autoimmune reactions, yet they can hinder anti-tumor immune responses, potentially exacerbating cancer progression. Elevated T-reg numbers and a low CD8+ T cell/T-reg ratio are often associated with poor prognosis in several cancer types, including melanoma, head and neck squamous cell carcinoma, ovarian cancer, and colorectal carcinoma [54].

The functional importance of T-regs in cancer has been demonstrated in murine models of melanoma, where the transient depletion of T-regs leads to enhanced anti-tumor immunity, improved tumor clearance, and prolonged survival. Numerous studies have shown an increased presence of T-regs in the peripheral blood of patients with metastatic melanoma compared to healthy individuals of a similar age. In addition, T-regs are highly concentrated within the tumor microenvironment of melanoma patients, including primary lesions, affected lymph nodes, and metastatic sites, where they exert potent immunosuppressive effects [55].

In this context, the anti-T-reg action carried out by NTZ may have beneficial effects in patients with melanomatous skin lesions.

### 4.2. Anti CD20 mAbs

Anti-CD20 mAbs act as depletors of a specific blood class, the B cells, through various mechanisms, such as antibody-dependent cellular cytotoxicity, complement-dependent cytotoxicity, phagocytosis, and antibody-dependent cellular apoptosis, sparing B cell reconstitution capacity, pre-existing humoral immunity, and innate immunity [20]. OFA is indicated for adult patients with RR forms of MS with active disease as defined by clinical or imaging features, while OCR is approved for the treatment of adult patients with RRMS and PPMS [21].

The limited data available on skin malignancies in patients treated with OCR result from pivotal and open-label trials. These data allow a reflection on the role of CD20 in dermatological neoplastic lesions.

Several studies have reported significant amounts of infiltrating CD20+ cells in melanoma lesions [56,57,58,59,60]. However, although the role of B cells in melanoma has been extensively investigated, the results of studies have been conflicting, resulting in both pro- and anti-tumor functions. Some studies have shown that high percentages of both intratumoral and peritumoral B cell infiltrates correlate positively with both survival and metastasis rates [56,57]. Conversely, other studies found a correlation between poor prognosis and intra-tumoral CD20+ cell infiltration, or no significant correlation between these two parameters [59,60]. The conflicting results of some studies could be due to several factors, mainly the different stages of melanoma [61].

Several studies have shown that the tumor-promoting activity of sunlight in the skin is mediated by UV-induced DNA damage, the suppression of anti-tumor immune responses, and the promotion of subcutaneous inflammation. A more recent finding is that UV light suppresses immunity in mice by activating a specific subtype of regulatory B cells. Using a murine model of photo-carcinogenesis, Kok et al. showed that the depletion of UV-activated immunoregulatory B cells limits UV-induced skin tumor growth, improves survival and/or prevents metastasis to skin-draining lymph nodes. The researchers investigated the effect of anti-CD20 antibodies compared to placebo in mice exposed to increasing doses of UV radiation, before and after the appearance of UV-induced skin lesions [62]. They showed no prophylactic effect of B cell depletion before the appearance of skin lesions on UV-induced skin cancer, while the treatment of tumor-bearing mice with anti-CD20 antibodies significantly reduced tumor growth and metastasis.

In line with these findings, previous studies have shown that UV-activated B cells suppress T cell-driven immune responses, in part by interfering with dendritic cells and producing immunoregulatory IL-10 [63]. Kok et al., with their studies, ascertained the therapeutic efficacy of anti-CD20 mAbs in mice with UV-induced skin tumors, raising the possibility of using the same strategy in humans upon demonstration that the results obtained in mice hold true for humans [62].

### 4.3. Alemtuzumab

ALZ exerts its effect by binding to the surface antigen CD52 expressed on lymphocytes and other white blood cells, causing cell lysis by a variety of mechanisms. In the European Union, it is approved for use in adults diagnosed with active RRMS as defined by clinical or imaging features [36,37]. While it is known that CD52 expression in chronic lymphocytic leukemia and breast cancer has prognostic and predictive value, its significance in melanoma is unclear [64,65,66]. Little is known about the role of CD52 inhibitors in skin tumors. Luka de Vos-Hillebrand and colleagues investigated the prognostic and predictive significance of CD52 mRNA expression in melanoma, examining its impact on prognosis, response to an immune checkpoint blockade (ICB), and elements of the tumor microenvironment (TME) [67]. Their studies showed that CD52 mRNA is expressed in a small subset of melanoma cells that co-express the immune checkpoint and that CD52 expression correlates with specific response characteristics to ICB in melanoma. Therefore, the use of anti-CD52 as an ALZ requires caution in patients receiving concurrent therapy with immune checkpoint inhibitors [67].

Therefore, it appears from this discussion that the targets of mAbs may have a localization or involvement of skin cells as well, and therefore it could be conceivable that mAbs could also impact the skin. However, despite the data available in the literature, the debate on a possible association between cutaneous malignancies and MS therapy with mAbs is still open, because there is a lack of both long-term clinical data and more extensive preclinical laboratory data.

However, this literature review offers significant strengths that may be helpful in gaining insight and shedding light on this topic. Firstly, a comprehensive review of the literature that focuses not on DMTs with an already known risk of dermatological malignancy, but on mAbs, which are gaining an increasingly prominent role in MS treatment; by drawing information from existing trials and clinical data, it is possible to obtain a broad overview on the incidence of malignancies in pwMS treated with mAbs. The selection of data and information provided by neurologists and dermatologists can also increase the credibility and depth of the review, leading to more reliable conclusions. Another strength lies in the potential to draw attention to malignancies associated with specific mAbs, with useful implications for clinical practice and appropriate patient management. Finally, by highlighting gaps in the current literature, the review may direct future research efforts to critical areas that require further investigation (Table 2).

A limitation of this review is the lack of both more extensive preclinical data and long-term clinical studies on the incidence of malignancy in pwMS treated with mAbs, which prevents us from drawing definitive conclusions on this issue. In addition, the heterogeneity of study designs, patient populations, and treatment regimens in the existing literature may complicate data synthesis and affect the reliability of results. Another limitation is the risk of potential bias: people with disabilities were often treated with previous multiple medications (including non-mAbs), which makes it difficult to selectively identify the risk associated with specific mAbs. In addition, other risk factors for the development of skin cancer, such as age, personal and family history of skin cancer, and actinic damage, need to be taken into account (Table 2).

Opportunities arising from this review include an increased awareness of this issue, which may highlight the need for dermatological assessment prior to mAb treatment in patients with disabilities and for an individualized dermatological follow-up program based on personal risk profiles. Furthermore, the results of this review may encourage the implementation of a multidisciplinary approach in clinical practice, including collaboration between dermatologists and neurologists, and stimulate interdisciplinary research to better understand the effects of mAb treatment (Table 2).

A potential threat is the emergence of new therapies for MS and a consequent reduction in the attention paid to mAbs and consequently, to the results of the review (Table 2).

## 5. Conclusions

Although the immune system and its regulation may play a crucial role in the development, progression, or metastasis of a skin malignancy, the currently available data do not allow us to establish a clear link between treatment with mAbs and the risk of skin cancer. The variability of the results could depend on several factors beyond the action on a specific target, such as the drug dose and the specific melanoma cell line.

The principal messages of this review draw attention to the need of dermatological evaluation before starting HETAs in pwMS, as these treatments are contraindicated in the presence of malignancy; it may encourage discussion between neurologists and dermatologists, both from a clinical and research perspective, in the management of pwMS undergoing immune therapy with mAbs. Furthermore, the dermatological follow-up program in patients receiving mAbs should be individualized and based on the risk profile of each individual, taking into account, among other factors, age, personal and family history of skin cancer, actinic damage, and the presence of other drugs that may increase risk.

Additionally, collaboration between neurologists and dermatologists should also be encouraged in the area of research. Further preclinical data and long-term clinical studies, as well as pharmacovigilance reports, should be encouraged to offer additional information to clarify the implications of long-term mAb treatment.

## Figures and Tables

**Table 1:** Skin malignancies reported in studies for each monoclonal antibody.

Monoclonal Antibody	Study	Skin Malignancies
NATALIZUMAB	AFFIRM [12]	1 metastatic melanoma
	TOP [13]	2 basal cell carcinomas, 2 melanomas in situ, 1 lentigo maligna, 1 choroidal melanoma 1 ocular melanoma
	SONAR [14]	137 melanomas (from FAERS) 7 melanomas (from peer-reviewed publications)
	RADAR [15]	205 melanomas (from FAERS) 78 melanomas (from EudraVigilance) 3 melanomas (from NMEDW)
	Castela et al. [16]	No changes in 248 pigmented lesions
	Pharaon et al. [17]	No changes in 775 melanocytic skin lesions
	Alping et al. [18]	2 melanomas
	TYGRIS [19]	10 basal cell carcinomas 13 melanomas
OCRELIZUMAB	OPERA [22]	1 melanoma in EP: 1 melanoma and 2 basal cell carcinoma
	ORATORIO [23]	3 basal cell carcinomas in EP: 1 squamous-cell carcinoma and 1 basal cell carcinoma
	CASTING [24]	1 basal cell carcinoma
	Smoot et al. [26]	4 basal cell carcinomas 1 melanoma
	CONFIDENCE [27]	3 melanomas, 2 basal cell carcinoma, 1 squamous cell carcinoma of the skin
OFATUMUMAB	ASCLEPIOS [34]	2 basal cell carcinomas 1 melanoma
ALEMTUZUMAB	CARE-MS I–II [38,39,40]	6 basal cell carcinomas 4 melanomas
	CAMMS03409 and TOPAZ [44,46]	2 melanomas

Legend: EP: open-label extension phase.

**Table 2:** SWOT analysis of the literature review of dermatological malignancies during treatment with mAbs for MS.

Strengths	Weaknesses
Comprehensive data collection: the review can leverage existing studies and clinical data, providing a broad understanding of the incidence of malignancy in patients treated with mAbs for MS. Expert collaboration: involvement of neurologists and dermatologists can enhance the credibility and depth of the review, leading to more reliable conclusions. Increased attention to malignancies associated with specific mAbs: useful implications for clinical practice and appropriate patient management. Guidance for future research: highlighting gaps in the current literature, the review can direct future research efforts towards critical areas needing further exploration.	Limited longitudinal data: the review may face challenges due to the lack of more extensive preclinical data and long-term clinical studies on the incidence of malignancy, which can limit the ability to draw definitive conclusions. Heterogeneity of studies: variability in study designs, patient populations, and treatment regimens across the existing literature can complicate the synthesis of data and affect the reliability of findings. Potential bias: pwMS have often been treated with previous multiple medications (including non-mAbs), which may pose a limitation in selectively identifying the risk associated with specific mAbs. Additionally, other risk factors for the development of cutaneous malignancies should be considered, such as age, personal and family history of skin cancer, and actinic damage.
Opportunities	Threats
Increased awareness: the review can highlight the need for a dermatological evaluation before starting mAbs in pwMS; the dermatological follow-up program in patients receiving mAbs should be individualized, based on the risk profile of each individual. Clinical multidisciplinary collaboration: findings from the review may encourage multidisciplinary approach in clinical practice, including dermatological-neurological collaboration. Interdisciplinary research: the review can foster interdisciplinary research to better understand the implications of mAb treatment.	Other treatments: as new therapies emerge for MS, the focus on mAbs may diminish, potentially reducing interest in the review’s findings.
